# Infectious and inflammatory disorders might increase the risk of developing idiopathic intracranial hypertension – a national case-control study

**DOI:** 10.1177/0333102420928079

**Published:** 2020-05-25

**Authors:** Anna Sundholm, Sarah Burkill, Elisabet Waldenlind, Shahram Bahmanyar, A Ingela M Nilsson Remahl

**Affiliations:** 1 Department of Clinical Neuroscience, Karolinska Institutet and Department of Neurology, Karolinska University Hospital, Sweden; 2 Centre for Pharmacoepidemiology, Department of Medicine, Karolinska Institutet, Solna, Sweden; 3 Saw Swee Hock School of Public Health, National University of Singapore, Singapore; 4 Centre for Pharmacoepidemiology, Department of Medicine, Solna, Karolinska Institutet and Centre for Psychiatry Research, Karolinska Institutet, Sweden

**Keywords:** Pseudotumor cerebri syndrome, benign intracranial hypertension, risk factors, inflammation, infection, case-control study

## Abstract

**Objective:**

To investigate whether conditions causing inflammatory activation are associated with increased risk of idiopathic intracranial hypertension.

**Methods:**

All newly diagnosed idiopathic intracranial hypertension patients (cases) in Sweden between 2000–2016 were identified using pre-determined algorithms (n = 902) and matched with five controls from the general population and five individuals with an obesity diagnosis (n = 4510) for age, sex, region, and vital status. National health registers provided information on infections, inflammatory disorders and dispensed medications. Conditional logistic regression was used to estimate odds ratios and 95% confidence intervals.

**Results:**

Compared to general population controls, the cases had fourfold increased odds of having an infection (odds ratio = 4.3, 95% confidence interval 3.3–5.6), and threefold increased odds of an inflammatory disorder the year prior to idiopathic intracranial hypertension diagnosis (odds ratio = 3.2, 95% confidence interval 2.4–4.3). Organ specific analyses showed that odds were increased for the study diseases in the respiratory organ, kidney organ and gastrointestinal tract, but not for female genital infections. Similar results were found when comparing idiopathic intracranial hypertension with obese controls though the odds ratios were of lower magnitude. Sub-analyses on exposure to anti-infectious and anti-inflammatory drugs confirmed the increased odds ratios for idiopathic intracranial hypertension patients.

**Conclusions:**

These findings suggest that major inflammatory activation may be a risk factor in idiopathic intracranial hypertension development.

## Background

Idiopathic intracranial hypertension (IIH) is a disorder that induces symptoms such as headache, visual disturbances and pulsatile tinnitus due to increased intracranial pressure (ICP) of unknown cause (1). The incidence varies between nations but metanalyses with pooled results report the incidence to be 1.2 per 100,000 individuals in the adult population (2). It has been reported to be slightly lower in Sweden (0.65 per 100,000) (3). IIH is most frequent among obese females of childbearing age. Many risk factors have been proposed to be associated with IIH; for example, obesity, exposure to tetracyclines, withdrawal from corticosteroids, kidney failure, anaemia, hypervitaminosis A, Addison’s disorder and hyperparathyroidism. However, the findings are not conclusive, and the pathogenesis remains unknown (4,5). One hypothesis has addressed the possibility that an inflammatory response contributes to IIH pathogenesis (6–8). Obesity is a disorder known to cause a chronic pro-inflammatory state in the body (9–11), and it is strongly associated with IIH development. Recent weight gain prior to diagnosis is also common (12). However, other risk factors must also be involved in IIH development and dysregulation of ICP in the brain since most patients with obesity do not develop IIH. Several case reports describe, mostly in children, a clinical syndrome like IIH but with its onset in association with syphilis, hepatitis A, varicella, measles, or other viral infections (13–18).

Several mechanisms have been proposed by which an inflammatory process could be involved in IIH pathogenesis, partly to explain the strong association of obesity and IIH. One potential pathway relates to increased activity of the enzyme 11β-hydroxysteroid dehydrogenase type 1 (11β-HSD1), which has been shown to be dysregulated with increased activity in both obesity and IIH (6). In humans, 11β-HSD1 increases local cortisol levels. Long-standing high cortisol levels have been shown to increase secretion of pro-inflammatory mediators (19) and possibly to increase the production of cerebrospinal fluid by affecting sodium transporters in the choroid plexus (6). Weight loss and lower ICP values has been shown to reduce 11β-HSD1-levels in IIH patients (20). A second hypothesis has discussed the possible role of proinflammatory cytokines and apokines such as leptin, IL-2, IL-10, IL-12, IL-17, and TNF-α. Several studies have demonstrated increased or decreased levels of these inflammatory mediators indicating that pro-inflammatory activation could plausibly be involved in the pathogenesis of IIH (8,21–24).

The aim of this nationwide case-control study was to further test the inflammatory hypothesis via investigating the possible association between IIH and exposure to infectious or inflammatory disorders during the year prior to IIH diagnosis. We also investigated the association for exposure to anti-infectious and anti-inflammatory drugs.

## Methods

### Study population, study design and outcome

This is a retrospective national population-based case-control study. Adult patients (aged 18 and older) in Sweden with an incident IIH diagnosis between 2000 and 2016 (using the International Statistical Classification of Diseases and Related Health Problems – Tenth Revision (ICD-10) code G93.2) were eligible.

A validation study was previously performed to investigate to what extent a diagnosis code G93.2 in the Swedish National Patient Register (NPR) also fulfilled the clinical diagnosis criteria for IIH according to the modified Dandy Criteria (3). The results indicated that only 65% of cases in NPR were true IIH patients. We therefore developed two algorithms that better predicted which patients were correctly diagnosed (using the parameters: Age, recorded diagnosis code G93.2 three or more times, and in a second algorithm also including treatment with acetazolamide). The algorithms improved the prediction of which patients to include to 86–88% positive predictive value (including data from Prescribed Drug Register (PDR) slightly improved the PPV) (25)

The final study sample comprised all patients who had received the diagnosis code G93.2 in Sweden in the NPR between 2000 and 2016 and were identified as being predicted as correctly diagnosed with IIH according to either of our two algorithms. Patients were excluded if they had received a diagnosis of IIH prior to the year 2000.

Each case was matched with five controls from the general population (GP) and five obese controls from NPR with a reported ICD-10 code E66, none of whom had a registered IIH diagnosis. The matching factors were age, sex, region and vital status on the index date; that is, the date of the very first recorded IIH diagnosis for each individual IIH patient.

### Exposures

ICD-10 diagnosis codes in the NPR were used to identify whether individuals had been diagnosed with infections or inflammatory disorders in the year prior to index date (see Supplemental Table 1 for list of codes). Regardless of number of codes, the individuals were counted as exposed. To receive sufficient sample size to analyse infections and inflammatory disorders, the diagnosis codes were grouped as exposure to: a) all infectious and/or inflammatory disorders; b) an infectious disorder; c) an inflammatory disorder; d) infectious/inflammatory disorders by affected organ; e) specific diagnosis groups. 

Since primary care does not report to the NPR, we also included data from PDR on anti-inflammatory and anti-infectious treatments to also capture less severe infectious and inflammatory conditions that frequently are diagnosed and treated in the primary care settings. This secondary analysis using dispensed medications was used as a proxy for exposure to inflammatory and infectious disease . Included patients for the study of medication dispensations were those with a first IIH diagnosis between July 2006 and December 2016 and their matched controls. This analysis considered the odds of developing IIH for those collecting a prescription for anti-inflammatory or anti-infectious purposes. We also did sensitivity analyses on exposure to inflammatory and infectious disorders in the time period 1–3 years prior to index date.

### Register information

The registries used were the NPR (26,27), the PDR (28) and the Swedish Total Population Register (29). The registers were linked using the unique personal identification number issued to all Swedish residents at birth or immigration (30). The NPR includes data on age, sex, date of admission and discharge, hospital, clinic, the main diagnosis and up to 21 secondary diagnoses, data on medical procedures with national coverage since 1997 (27), and secondary healthcare contacts since 2001 (excluding primary care). The NPR has generally good coverage of diagnoses in specialised inpatient and outpatient settings; however, validity of the coding differs depending on the diagnosis. In general, the NPR has shown good validity of registered diagnoses with a PPV of between 85–95% depending on the disease (26); information specifically on infectious and inflammatory disorders is not available. The proportion of missing main diagnosis from the NPR is around 1% (27). The PDR started recording data on dispensed prescriptions from July 2005. All prescribed medications collected from the pharmacies in Sweden are recorded in the PDR. Over the counter medications and treatments administered in hospitals are however not identifiable in this register. Information is available on date of prescription and dispensation, and on the Anatomic Therapeutic Chemical (ATC) classification of the drug prescribed. We used ATC code information to identify prescriptions of drugs intended to treat inflammation or infection (see Supplemental Table 1). Health care registers in Sweden are generally regarded as having high quality and cover all inhabitants in Sweden. The Total Population Register holds demographic information on Swedish residents, including data on region of residence, migration, date of death, and educational attainment. The Total Population Register has an extensive cover of births and deaths (almost 100%) and immigration and emigration (90–95%), usually recorded within 30 days of the event occurring (29).

### Ethics statement

The study is approved by the Ethical Committee in Stockholm county. Ethics approval was also granted from the register holders.

### Statistics

Conditional logistic regression was used to estimate odds ratios (OR) and 95% confidence intervals (CI) comparing IIH to GP controls as well as comparing IIH to obese controls. This model assumes clustering within the matched groups and the variance is adjusted accordingly. The frequency was reported. The adjusted model included educational level (categorised as level 1: ≤ 9 years of compulsory school, level 2: > 9 year of compulsory school and ≤ high school, level 3: > higher education after high school) as a proxy for socioeconomic status. STATA 12 was used to undertake the statistical analyses.

## Results

Between 2000 and 2016, 1439 patients were identified as having received an incident IIH diagnosis in the NPR. Before applying the algorithms, the sex distribution was F|M: 77% versus 23%, mean age 38.6, and median age 36. The algorithms identified 902 patients predicted to be correctly diagnosed as IIH patients (see Table 1 for demographic characteristics). Educational level was more similar among IIH and obese controls, with 17–18% achieving 9 years or less of compulsory school compared to 10% among the GP controls as their highest educational attainment. Almost 50% of GP controls attained a higher education qualification (university) compared to approximately 30% of IIH and obese controls.

**Table 1.** Characteristics of the cases and controls.

	IIH	GP controls	Obese controls
Participants n	902	4510	4510
Age at diagnosis/index date (years):			
Mean (SE)	32.2 (0.17)	32.2 (0.17)	32.2 (0.17)
Median (IQR)	29 (23–38)	29 (23–38)	29 (23–38)
Female n (%)	767 (85.0)	3835 (85.0)	3835 (85.0)
Mean age (SE)	31.5 (0.11)	31.5 (0.11)	31.5 (0.11)
Median age (years) (IQR)	29 (23–38)	29 (23–38)	29 (23–38)
Male n (%)	135 (15.0)	675 (15.0)	675 (15.0)
Mean age (SE)	36.2 (0.35)	36.2 (0.35)	36.2 (0.35)
Median age (years) (IQR)	33 (24–47)	33 (24–47)	33 (24–47)
Information on achieved highest educational level n (%)	887 (98.3)	4454 (98.8)	4453 (98.7)
Compulsory school (≤9 years)	163 (18.4%)	439 (9.9%)	766 (17.2%)
Upper secondary (high school)	447 (50.4%)	1853 (41.6%)	2408 (54.1%)
University (level above high school)	277 (31.2%)	2162 (48.5%)	1279 (28.7%)

**Figure 1.**
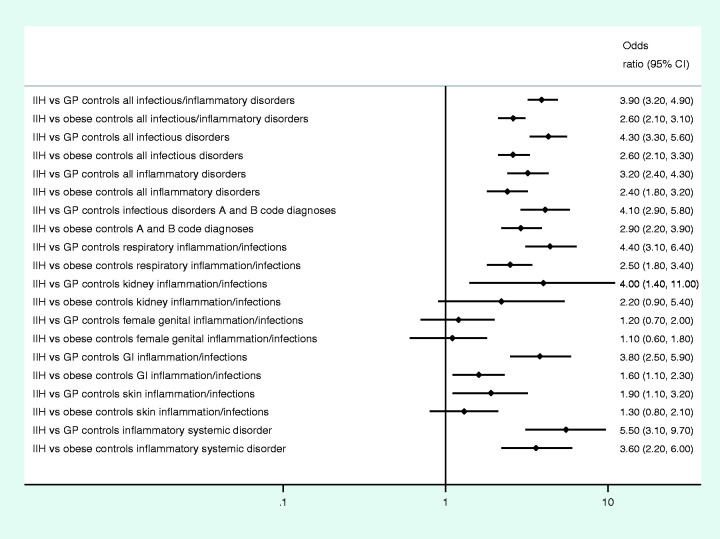
Odds ratio (adjusted for educational level) of registered diagnosis code in IIH patients compared to GP and obese controls. IIH: idiopathic intracranial hypertension cases; GP controls: matched general populations controls; obese controls: matched obese controls; GI disorders: gastrointestinal disorders.

The frequency of exposure to infections and inflammatory disorders was more common in IIH compared to controls (Table 2). For most infectious/inflammatory groups, a single contact with healthcare was most common in the year preceding the index date, while for inflammatory disorders (mostly chronic conditions) two or more contacts with healthcare were more common. There were increased odds of specialist clinic utilisation receiving diagnosis of infections, (OR_adjusted_ = 4.3, 95% CI 3.3–5.6) and inflammatory conditions (OR_adjusted_ = 3.2, 95% CI 2.4–4.3) the year prior to index date (Figure 1, crude OR and adjusted OR in Supplemental Table 2).

**Table 2.** Frequency of different exposures during the observation period in cases and controls.

Registered diagnosis code one year prior to index date (ICD-10 code)* and number of registered codes per individual	IIH (N = 902) n (%)	GP controls (N = 4510) n (%)	Obese controls (N = 4510) n (%)
All infectious or inflammatory disorders:			
Summary:	**179 (20)**	**258 (5.7)**	**397 (8.8)**
1	94 (10)	169 (3.8)	252 (5.6)
2	38 (4.2)	56 (1.2)	72 (1.6)
3+	47 (5.2)	33 (0.7)	73 (1.6)
All infectious disorders:			
Total exposed:	**121 (13)**	**148 (3.3)**	**252 (5.6)**
1	73 (8.1)	121 (2.7)	182 (4.0)
2	33(3.7)	16 (0.4)	45 (1.0)
3+	15 (1.7)	11(0.2)	25 (0.6)
All inflammatory disorders:			
Total exposed:	**80 (8.9)**	**127 (2.8)**	**177 (3.9)**
1	39 (4.3)	71 (1.6)	107 (2.4)
2	17 (1.9)	35 (0.8)	29 (0.6)
3+	24 (2.7)	21 (0.5)	41 (0.9)
Specific infectious disorders (A+B diagnosis codes):			
Total exposed:	**71 (7.9)**	**86 (1.9)**	**130 (2.9)**
1	48 (5.3)	74 (1.6)	99 (2.2)
2	17 (1.9)	6 (0.1)	20 (0.4)
3+	6 (0.7)	6 (0.1)	11 (0.2)
Respiratory inflammatory/infections:			
Total exposed:	**56 (6.2)**	**63 (1.4)**	**116 (2.6)**
1	34 (3.8)	47 (1.0)	86 (1.9)
2	13 (1.4)	13 (0.3)	18 (0.4)
3+	9 (1.0)	3 (0.1)	12 (0.3)
Kidney inflammation/infections:			
Total exposed:	**7 (0.8)**	**8 (0.2)**	**16 (0.4)**
1	5 (0.6)	8 (0.2)	15 (0.3)
2	1 (0.1)	0 (0)	1 (0.0)
3+	1 (0.1)	0 (0)	0 (0)
Female genital inflammation/infections:			
Total exposed:	**18 (2.4)**	**73 (1.9)**	**83 (2.2)**
1	13 (1.4)	64 (1.4)	66 (1.5)
2	4 (0.4)	9 (0.2)	15 (0.3)
3+	1 (0.1)	0 (0)	2 (0.0)
GI inflammation/infections:			
Total exposed:	**39 (4.3)**	**51 (1.1)**	**125 (2.8)**
1	22 (2.4)	33 (0.7)	78 (1.7)
2	5 (0.6)	7 (0.2)	27 (0.6)
3+	12 (1.3)	11 (0.2)	20 (0.4)
Skin inflammation/infections:			
Total exposed:	**20 (2.2)**	**53 (1.2)**	**78 (1.7)**
1	17 (1.9)	33 (0.7)	55 (1.2)
2	0 (0)	11 (0.2)	14 (0.3)
3+	3 (0.3)	9 (0.2)	9 (0.2)
Inflammatory systemic disorder:			
Total exposed:	**27 (3.0)**	**25 (0.6)**	**39 (0.9)**
1	8 (0.9)	8 (0.2)	10 (0.2)
2	7 (0.8)	11 (0.2)	9 (0.2)
3+	12 (1.3)	6 (0.1)	20 (0.4)

*Includes both repeated codes on the same diagnosis or different diagnoses including within the examined diagnostic group.

IIH: idiopathic intracranial hypertension; GP controls: matched general population controls; obese controls: matched obese controls.Bold figures are the summary of the numbers of having 1 code registered, 2 codes registered or 3 or more codes registered in the preceding year.

**Figure 2.**
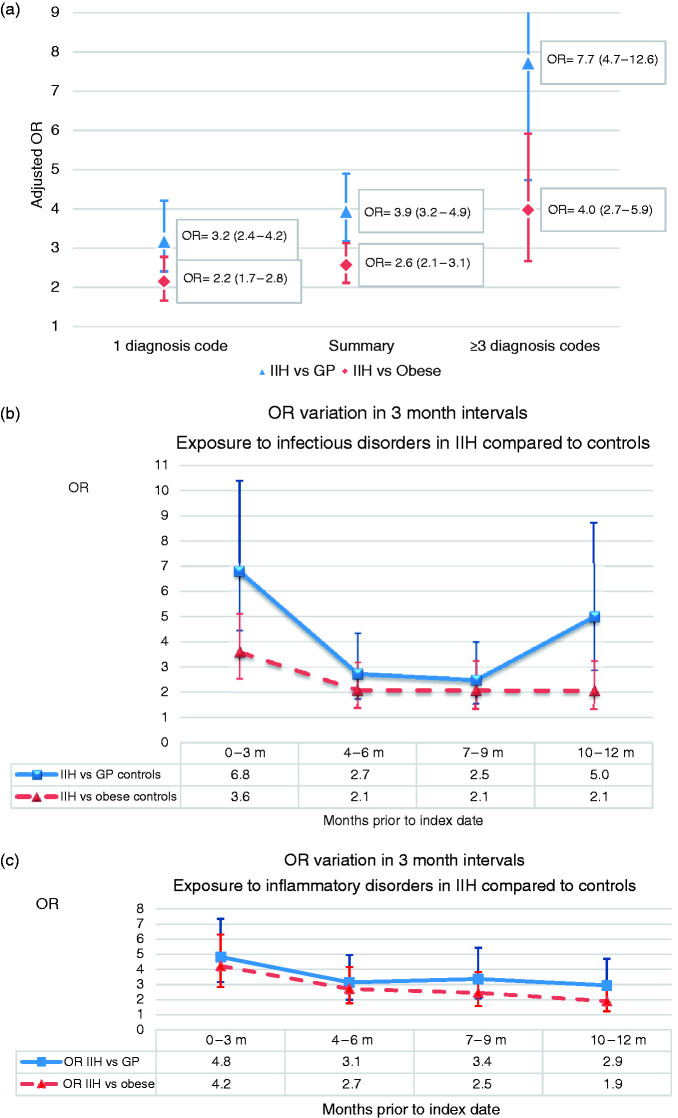
OR variation a) according to frequency of diagnosis codes for any infectious/inflammatory disorder b) on exposure to infectious disorders in 3-month intervals prior to index date, c) inflammatory conditions in 3-month intervals.Note: Summary = any number of infectious or inflammatory diagnosis codes registered.

Sensitivity analyses to evaluate frequency and time intervals of exposure were made. Receiving diagnosis codes several times (≥3) for any infectious or inflammatory disorder in the preceding year showed a much higher increased OR (OR = 7.7, 95% CI 4.7–12.6) for later developing an IIH diagnosis compared to GP controls, while only receiving one code increased odds by three (Figure 2(a)). Sub-analysis which considered different time periods in the year prior to IIH diagnosis separately (0–3, 4–6, 7–9 and 10–12 months) showed a particularly elevated OR for those diagnosed with an infectious or inflammatory disorder in the 3 months directly prior to IIH diagnosis. However, the OR for an IIH diagnosis among those given an infectious or inflammatory disorder diagnosis was elevated for all earlier 3-month time periods, although the magnitude of the effect was lessened (Figure 2(b), 2(c)). We did sensitivity analyses looking at exposure to infectious and inflammatory disorders during the time-period 3 years to 1 year prior to index date. The results for infectious disorders were OR_adjusted_ = 2.0, (95% CI 1.6–2.6) in IIH compared to GP controls and OR_adjusted_ = 1.1 (95% CI 0.9–1.4) compared to obese controls; for inflammatory disorders OR_adjusted_ = 2.7, (95% CI 2.1–3.6) compared to GP controls and OR_adjusted_ = 1.7 (95% CI 1.3–2.2) compared to obese controls.

The various infections/inflammations were sub-grouped according to organ involved or specific group of disorders. Compared to GP controls, adjusted odds were: For specific infections (all A+B ICD-10 diagnosis codes) OR_adjusted_ = 4.1 (95% CI 2.9–5.8), for systemic inflammatory illnesses OR_adjusted_ = 5.5 (95% CI 3.1–9.7). For infections/inflammatory disorders in the respiratory organ OR_adjusted_ = 4.4 (95% CI 3.1–6.4), kidney organ OR_adjusted_ = 4.0 (95% CI 1.4–11.4), and for gastrointestinal OR_adjusted_ =3.8 (95% CI 2.5–5.9). Exposure to female genital infectious/inflammatory disorders, however, were not associated with increased odds prior to development of IIH (Figure 1).

Compared to obese controls, the odds for having an inflammatory or infectious disorder the year prior to index date was statistically significantly increased among IIH patients (except for skin, female genital and kidney disorders), although the magnitude of the effect was lower than when the GP controls were used (Table 2, Figure 1, Supplemental Table 2).

**Figure 3.**
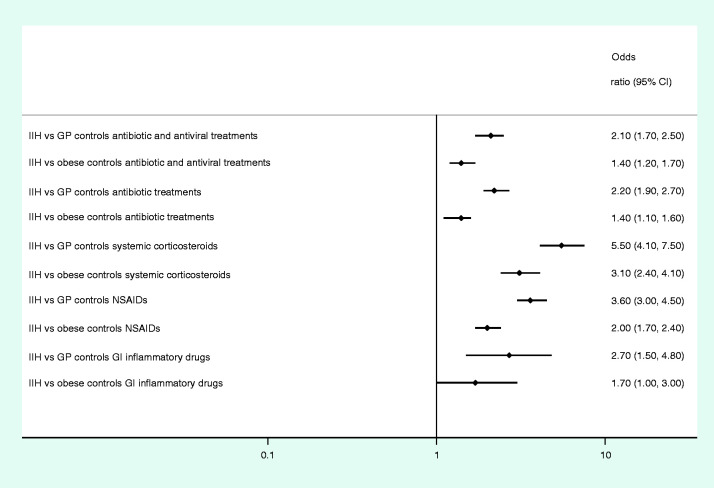
Odds ratio of dispensations from pharmacies 1 year prior to diagnosis (index date) in IIH patients compared to GP controls and compared to obese controls adjustments made for educational level. IIH: idiopathic intracranial hypertension cases; GP controls: matched general populations controls; obese controls: matched obese controls; GI anti-inflammatory drugs: gastrointestinal anti-infectious or anti-inflammatory drugs.

When analysing data from PDR, odds were statistically significantly increased (double to fivefold) of having been prescribed a treatment for infectious and inflammatory disorders for IIH patients compared with GP controls. Exposure to antibiotic treatments OR_adjusted_ = 2.2 (95% CI 1.9–2.7); to systemic corticosteroids, OR_adjusted_ = 5.5 (95% CI 4.1–7.5), and to non-steroidal anti-inflammatory drugs, OR_adjusted_ = 3.6 (95% CI 3.0–4.5) all showed increased risk of IIH development. The same applied when comparing IIH patients to obese controls, but the magnitude of the effect was less pronounced OR_adjusted_ =1.4–3.1 (Table 3, Figure 3, and Supplemental Table 3). We did sensitivity analyses on antibiotics by excluding tetracyclines, sulpha- and fluoroquinolone antibiotics (proposed risk factors to IIH). Our results were similar, although with a slightly (0.2) lower OR, (IIH vs GP: OR_adjused_ = 2.0, 95% CI 1.7–2.4 and IIH versus obese: OR_adjused_ = 1.2, 95% CI 1.0–1.4).

**Table 3.** Dispensation of medications for infectious and inflammatory disorders one year prior to index date.

Pharmacy withdrawn medication one year prior to index date	IIH patients N = 654 n (%)	GP controls N = 3270 n (%)	Obese controls N = 3270 n (%)
All anti-infectious treatments	276 (42)	832 (25)	1,126 (34)
All antibiotic treatments	259 (40)	720 (22)	1064 (33)
Corticosteroids for systemic use	97 (15)	103 (3)	178 (5)
Anti-inflammatory + antirheumatic non-steroidal drugs	213 (33)	363 (11)	644 (20)
Antidiarrheals, anti-inflammatory and anti-infectious local intestinal drugs	19 (2.9)	35 (1.1)	55 (1.7)

IIH: idiopathic intracranial hypertension; GP controls: matched general population controls; obese controls: matched obese controls.

## Discussion

This large population-based case-control study found significantly increased odds ratios of having received a diagnosis for infectious or inflammatory disorders in the year preceding the date of first IIH diagnosis. This association was verified by evaluating exposure to drugs prescribed to treat infections or inflammatory conditions. The increased odds ratios were more prominent if more frequent codes of infectious/inflammatory disorders had been received. To our knowledge, no previous nationwide study has investigated whether systemic infections or inflammatory conditions not affecting the central nervous system (CNS) are associated with increased risk of IIH.

Our findings suggest that an inflammatory activation could act as a possible contributory factor involved in the development of IIH. By investigating the number of diagnoses given in 3-month intervals prior to index date we evaluated if the results were stable or merely an artefact of surveillance bias (the IIH group might seek more healthcare the month prior to diagnosis by not feeling well). We found that there was an increased OR of infectious or inflammatory disorder in the months just prior to index date but otherwise there was a fairly stable OR during the time period investigated. There could be several explanations for this: The surveillance bias described above (resulting in higher identification and registration of infections) or our hypothesis that in fact a recent inflammatory activation works as the final trigger, increasing the intracranial pressure and thereby leading to a worsening of symptoms and a subsequent diagnosis. We also did sensitivity analyses looking at diagnoses of infectious disorders 1–3 years prior to index date and found no significance compared to obese controls, however exposure was more prevalent compared to GP controls. This could to some extent be explained by increased risk of infections in relation to obesity (10), as we know IIH patients to a large extent are obese. The odds of exposure to inflammatory disorders 1–3 years prior to diagnosis was still significantly increased compared to both control groups. This could be explained partly by inflammatory conditions, which many times are chronic disorders but could also indicate that low-grade inflammatory activation over a longer period of time could be a risk factor.

When subdividing infections and inflammatory disorders into specific infectious diseases or organ-oriented groupings, we found the most prominent differences between IIH and GP controls regarding specific infections, systemic inflammatory conditions, respiratory, gastrointestinal, and kidney conditions. Comparing IIH to obese controls, the effect sizes were smaller than compared to GP controls, and did not reach significance in kidney and skin disorders. However, there was no increased OR on exposure to female genital infectious/inflammatory disorders with either control group. Our findings do not appear to indicate that inflammation of a specific organ system is more prone to be associated with IIH than any other. However, it is plausible that disease severity and duration may be of importance given that the ORs of IIH development were highest among those who had a larger number of NPR records, denoting exposure to infection or inflammatory disorders.

We used individuals with an obesity diagnosis as a second control group. Since IIH patients to a large extent are obese, this analysis helps to control for factors related to obesity. Our results suggest that obesity has a partial confounding effect on our results since the effect size of increased OR were smaller. This may be due to obese persons being more prone to infections and several other chronic disorders (for example asthma), possibly related to the pro-inflammatory effect of obesity on the immune system (10). Our results indicate, however, that obesity does not fully explain the association between infectious and inflammatory disorders and IIH; rather, it may have a synergetic effect.

We observed that exposure to anti-inflammatory treatments or anti-infectious treatments is associated with IIH, consistent with our results using NPR data. It could be argued that the results on antibiotics are driven by previously reported risk factor drugs associated with IIH such as tetracyclines, sulfonamides and fluoroquinolone antibiotics (4,5,31). To investigate how this influenced our results, we performed sensitivity analyses on antibiotics by excluding tetracyclines, sulfonamides and fluoroquinolone antibiotics. Our results did not change materially. In a recent cohort study with controls on cycline antibiotics and IIH development, Eldweik et al. (32) reported a marginally increased risk; however, this was not significant after adjusting for confounding factors. The OR was slightly increased for antibiotic treatments in comparison with the obese controls, but when excluding risk factor antibiotics, it did not reach statistical significance. One explanation for this could be that obesity is a highly underestimated diagnosis code in registers and patients who do receive an obesity diagnosis in NPR are most likely severely obese and probably more prone to comorbidities and healthcare contacts compared to IIH patients.

IIH patients often present headache as one of their main symptoms. There are also IIH patients with increased ICP but without headache. When the ICP is normalised, the headache remains in more than half of patients, sometimes with more migraine and sometimes with more tension-type characteristics (33). Decreased pain thresholds caused by induced systemic inflammation has been proposed in humans by de Goeij et al. (34). Testing this is beyond the primary focus of this study but is an interesting pathophysiologic hypothesis for further studies.

### Strengths and limitations

The strengths of this study include that it is a national population-based design including all individuals with IIH diagnoses in Sweden over a long period (17 years), with subsequent filtering based on algorithms to improve the diagnostic validity of included cases. Therefore, although IIH is uncommon, a decent-sized study sample was obtained. IIH is almost exclusively diagnosed in the secondary care setting, meaning that most patients will be captured. However, some of the conditions that we investigated as possible risk factors are also rare, which reduces statistical power. To increase statistical power of the study, exposures (diseases/treatments) had to be analysed in subgroups. In the main analyses we investigated infectious disorders and inflammatory disorders as two separate groups.

Often patients report having had symptoms several months before finally getting their IIH diagnosis. Therefore, a time interval of 1 year was considered appropriate. If and for how long exposure to risk factors could play a role for disease development is not known.

Another limitation is that some chronic inflammatory disorders may have been underestimated if the study participants did not have contact with secondary healthcare on an annual basis. The exposure period of 1 year prior to index date was chosen to allow investigation of a hypothesised association between acute exacerbation/active inflammatory disease and association with diagnosis of IIH. However, we also reported a sensitivity analysis looking at 1–3 years before index date.

CNS infections with increased cell counts in the cerebrospinal fluid can also cause increased intracranial hypertension with similar symptoms to IIH and they might require similar treatment with pressure-lowering substances, but are diagnosed with secondary intracranial hypertension (sIH). When developing the algorithms used in this study (25) our results showed that the algorithms were weak at separating sIH patients from true IIH cases. Given the limitations of the algorithms, we were not able to study whether CNS infections/inflammations (G0 diagnosis) were specifically increasing the risk of IIH, because those with CNS infections are more likely to be incorrectly identified by the algorithm, and in fact should have been classified as sIH. Sensitivity analyses excluding CNS infections (ICD-10 A17 code and tuberculosis (ICD-10 code A39) did not change our results materially.

The comparison with an obese control group was an important part of the study and has provided insight into a potential shared mechanism for IIH and obesity. There may, however, be a selection bias by comparing IIH cases with controls having more extreme obesity and possibly more health problems due to comorbidities.

Another limitation is the lack of primary care data. Therefore, disorders more commonly diagnosed in primary care such as many common infections and inflammatory respiratory illnesses (asthma, chronic obstructive lung disease), are less prevalent in our material than expected. To address this limitation, data from PDR of dispensed medications used to treat infectious and inflammatory disorders were used as a proxy for these disorders to assess whether results were similar. Finally, we did not have information about the specific indication for prescribing these drugs. Another limitation is the lack of data on over-the-counter medication, which especially affects NSAID use.

Our results only describe the Swedish population; however, similar results could be expected in settings with a comparable range of diseases and living conditions.

## Conclusion

Exposure to an infectious or inflammatory disorder in the year preceding IIH diagnosis was significantly associated with increased odds of developing IIH. The findings support the hypothesis that a major inflammatory activation could act as a trigger factor to IIH development. The possible mechanisms for this need further investigation.

## Article highlights

- We observed that exposure to infectious and inflammatory disorders were 3–4 times more common the year prior to IIH diagnosis compared to population controls.
- This difference remained, however, lower amplitude (2.5 times more common) when comparing to matched controls with an obesity diagnosis, even though the obese control group were expected to be more severely obese compared to IIH cases and thereby probably more prone to comorbidities.
- Inflammatory activation may be an important risk factor valuable to investigate when considering pathophysiology processes behind IIH.

## Supplemental Material

sj-pdf-1-cep-10.1177_0333102420928079 - Supplemental material for Infectious and inflammatory disorders might increase the risk of developing idiopathic intracranial hypertension – a national case-control study Click here for additional data file. Supplemental material, sj-pdf-1-cep-10.1177_0333102420928079 for Infectious and inflammatory disorders might increase the risk of developing idiopathic intracranial hypertension – a national case-control study by Anna Sundholm, Sarah Burkill, Elisabet Waldenlind, Shahram Bahmanyar and A Ingela M Nilsson Remahl in Cephalalgia

## Statistical analyses

Statistical analyses were undertaken by Sarah Burkill, Karolinska Institutet and Anna Sundholm, Karolinska Institutet.
