# Supplementary material for: Infectious and inflammatory disorders might increase the risk of developing idiopathic intracranial hypertension – a national case-control study
Source: Cephalalgia. 2020 May 25;40(10):1084–94. doi: 10.1177/0333102420928079 (PMC7457460; doi:10.1177/0333102420928079)
Supplement: sj-pdf-1-cep-10.1177_0333102420928079 - Supplemental material for Infectious and inflammatory disorders might increase the risk of developing idiopathic intracranial hypertension – a national case-control study [file sj-pdf-1-cep-10.1177_0333102420928079.pdf]

Supplementary table 1:

ICD-10 codes used for the analyses:	
**All infectious and inflammatory disorders:**	All A+B diagnosis, J00-22, L00-08, N10, N12, N70-71, K35, K81, K85, M00-03, J40-46, N00-01, N03, N05, N11, M05-14, M30-36, K50-52, K90, L20-30
**Infectious disorders:**	All A+B diagnosis, J00-22, L00-08, N10, N12, N70-71, K35, K81, K85, M00-03
**Inflammatory disorders:**	J40-46, N00-01, N03, N05, N11, M05-14, M30-36, K50-52, K90, L20-30
**Specific infections:**	A + B diagnosis
**Respiratory inflammation/infections:**	J00-22, J40-46
**Kidney inflammation/infections:**	N00-01, N03, N05, N10-12
**Female genital inflammation/infections:**	N70-77
**Systemic inflammatory disorder:**	M30-36, M05-14
**GI inflammation/infections:**	K20, K29, K50-52, K35-37, K61, K73, K75, K80-81, K85, K90
**Skin inflammation/infections:**	L00-08, L20-30

ATC codes used for the analyses:	
**Antibiotic+antiviral treatments:**	J01, J05, D06, P
**Antibiotic treatments**	J01
**Systemic corticosteroids:**	H02
**Non-steroidal antiinflammatory drugs:**	M01
**GI drugs:**	A07

Supplementary Table 2: Odds ratio of registered diagnosis code in IIH patients compared to controls.

**Type of exposures/disorders included:**	**Comparison between:**	**OR_{crude} (95% CI)**	**$OR_{adjusted}$* (95% CI)**
**All infectious or inflammatory disorders:**	**IIH vs GP controls**	**4.2 (3.4-5.2)**	**3.9 (3.2-4.9)**
	IIH vs obese controls	2.6 (2.1-3.1)	2.6 (2.1-3.1)
**All infectious disorders:**	**IIH vs GP controls**	**4.7 (3.6-6.1)**	**4.3 (3.3-5.6)**
	IIH vs obese controls	2.6 (2.1-3.3)	2.6 (2.1-3.3)
**All inflammatory disorders:**	**IIH vs GP controls**	**3.3 (2.5-4.4)**	**3.2 (2.4-4.3)**
	IIH vs obese controls	2.4 (1.8-3.2)	2.4 (1.8-3.2)
**Infectious disorders (A+B diagnosis codes):**	**IIH vs GP controls**	**4.6 (3.3–6.4)**	**4.1 (2.9–5.8)**
	IIH vs obese controls	2.9 (2.2–3.9)	2.9 (2.2–3.9)
**Respiratory inflammation/infections:**	**IIH vs GP controls**	**4.6 (3.2–6.6)**	**4.4 (3.1–6.4)**
	IIH vs obese controls	2.5 (1.8–3.4)	2.5 (1.8–3.4)
**Kidney inflammation/infections:**	**IIH vs GP controls**	**4.4 (1.6–12.1)**	**4.0 (1.4–11.4)**
	IIH vs obese controls	2.2 (0.9–5.3)	2.2 (0.9–5.4)
**Female genital inflammation/infections:**	**IIH vs GP controls**	**1.2 (0.7–2.1)**	**1.2 (0.7–2.0)**
	IIH vs obese controls	1.1 (0.7–1.8)	1.1 (0.6–1.8)
**GI inflammation/infections:**	**IIH vs GP controls**	**3.9 (2.6–6.0)**	**3.8 (2.5–5.9)**
	IIH vs obese controls	1.6 (1.1–2.3)	1.6 (1.1–2.3)
**Skin inflammation/infections:**	**IIH vs GP controls**	**2.0 (1.1–3.3)**	**1.9 (1.1–3.2)**
	IIH vs obese controls	1.3 (0.8–2.1)	1.3 (0.8–2.1)
**Inflammatory systemic disorder:**	**IIH vs GP controls**	**5.5 (3.2–9.6)**	**5.5 (3.1–9.7)**
	IIH vs obese controls	3.6 (2.2–5.9)	3.6 (2.2–6.0)

OR= odds ratio, 95% CI= 95% confidence interval, IIH = idiopathic intracranial hypertension cases, GP controls = matched general populations controls, obese controls = matched obese controls, GI disorders= gastrointestinal disorders. $OR_{adjused}$*= OR adjusted for educational level

Supplemental Table 3: Odds ratio of dispensations from pharmacies one year prior to diagnosis (index date)

**Type of exposures/medications included:**	**Comparisson between:**	**OR_{crude} (95% CI)**	**$OR_{adjusted}$* (95% CI)**
**Antibiotic+antiviral treatments**	**IIH vs GP controls**	**2.2 (1.8–2.6)**	**2.1 (1.7–2.5)**
	IIH vs obese controls	1.4 (1.2–1.7)	1.4 (1.2–1.7)
**Antibiotic treatments**	**IIH vs GP controls**	**2.3 (2.0–2.8)**	**2.2 (1.9–2.7)**
	IIH vs obese controls	1.4 (1.1–1.6)	1.4 (1.1–1.6)
**Systemic corticosteroids**	**IIH vs GP controls**	**5.5 (4.1–7.5)**	**5.5 (4.1–7.5)**
	IIH vs obese controls	3.1 (2.4–4.0)	3.1 (2.4–4.1)
**Non-steroidal antiinflammatory drugs**	**IIH vs GP controls**	**3.9 (3.2–4.7)**	**3.6 (3.0–4.5)**
	IIH vs obese controls	2.0 (1.7–2.4)	2.0 (1.7–2.4)
**GI antiinflammatory drugs**	**IIH vs GP controls**	**2.7 (1.6–4.7)**	**2.7 (1.5–4.8)**
	IIH vs obese controls	1.8 (1.0–3.0)	1.7 (1.0–3.0)

OR= odds ratio, 95% CI= 95% confidence interval, IIH = idiopathic intracranial hypertension cases, GP controls = matched general populations controls, obese controls = matched obese controls, GI antiinflammatory drugs= gastrointestinal anti-infectious or anti-inflammatory drugs. $OR_{adjused}$*= OR adjusted for educational level
